# Love Forgiveness and Subjective Well-Being in Chinese College Students: The Mediating Role of Interpersonal Relationships

**DOI:** 10.3389/fpsyg.2021.634910

**Published:** 2021-06-04

**Authors:** Tianyi Cheng, Qiyi Lin, Hong Fu

**Affiliations:** ^1^School of Psychology, Nanjing Normal University, Nanjing, China; ^2^School of Educational Science, Huaiyin Normal University, Huai’an, China

**Keywords:** Chinese college students, love forgiveness, interpersonal relationships, subjective well-being, the mediation effect

## Abstract

Love forgiveness is categorized as forgiveness in a specific relationship, which is the tendency of individuals to forgive the objects of their interpersonal relationships. We investigated 831 undergraduate students in China with a love forgiveness questionnaire, a subjective well-being questionnaire and an interpersonal relationship comprehensive diagnostic, including demographic variables. Students of different genders and grades showed significant differences across the three questionnaires. There were significant correlations between love forgiveness, interpersonal relationships, and subjective well-being of Chinese college students. Interpersonal relationships played an intermediary role between love forgiveness and subjective well-being of Chinese college students. Specifically, whereas Chinese college students’ love forgiveness could directly promote the improvement of subjective well-being, love forgiveness could also indirectly improve subjective well-being through interpersonal relationships. The mediating effect of interpersonal relationships between love forgiveness and subjective well-being accounted for 40.52% of the total effect. This provides a new way of thinking for psychological counselors to approach the intimate relationship problems of college students.

## Introduction

Forgiveness has attracted attention from scholars in recent years as a new research subject of positive psychology. Forgiveness can be defined as a reduction in avoidance and revenge motivation accompanied by a change in prosocial motivation from an individual toward an offender following an interpersonal conflict (McCullough et al., 2006). Forgiveness is divided into trait forgiveness, forgiveness in a specific relationship, and forgiveness in a situation (Zhang and Fu, 2014). Forgiveness in love and in marriage is categorized as forgiveness in a specific relationship, which is the tendency of individuals to forgive the objects of their specific interpersonal relationships (Paleari et al., 2009; Zhang and Fu, 2014). Presently, research on the forgiveness of specific relationships in Western countries mainly focuses on marital relationships and rarely involves non-marital romantic relationships. In China, there are only a few studies on forgiveness in marital relationships and non-marital romantic relationships. We present love forgiveness as a tool to assess forgiveness in non-marital relationships that may help to close the gap in research in this area.

Subjective well-being is one of the most important factors associated with forgiveness. In China, the research of Liu and Wu (2011) and Chan (2013) has shown that forgiveness has a direct impact on subjective well-being, which is consistent with the research results in western countries (Bono et al., 2008; Tse and Yip, 2009). In addition, both studies of Western and Eastern cultural contexts showed that subjective well-being has a predictive effect on forgiveness as well (Xu, 2012; Yárnoz-Yaben et al., 2016). Thus, forgiveness in intimate relationships, and by extension love forgiveness, may also be related to subjective well-being.

Interpersonal relationships are often discussed as an additional factor associated with forgiveness. Interpersonal relationship refers here to a personal relationship in the general life of an individual. Studies in China have shown that forgiveness helps individuals improve and maintain their relationships, with higher levels of forgiveness associated with better relationships among college students (Li et al., 2010; Luo et al., 2012). This research result is also supported by studies in the context of Western culture (Paleari et al., 2005). Karremans et al. (2003) claims that forgiveness could improve a person’s subjective well-being by maintaining and repairing intimate relationships; that is, interpersonal relationships play an intermediary role between forgiveness and subjective well-being. The research in China of Jiang and Tan (2016) supports this view.

Subjective well-being is the latent variable. However, the research of Jiang and Tan (2016) used Causal Steps Approach, namely regression, to test subjective well-being as explicit variables, which may lead to errors. The relationship between forgiveness, interpersonal relationship and subjective well-being among Chinese college students still needs to be tested. In addition, whether the relationship between love forgiveness and these two factors (interpersonal relationships and subjective well-being) has similar correlations has not been studied no matter in eastern countries or western countries. From the perspective of the competitive model, the role of interpersonal relationship between love forgiveness and subjective well-being may be a moderating effect or a mediating effect. According to the point of view of Karremans et al. (2003) and Jiang and Tan (2016), and consider it from a theoretical perspective, we inferred that interpersonal relationships may mediate between love forgiveness and subjective well-being.

According to the White Paper on Chinese College Students’ Love in 2016 released by China Love Growth Platform, China University Youth Leaders Summit, and Peking University Yanyuan Bossi Psychological Counseling Center, 80% of the 35.59 million Chinese students on campuses had been in an intimate relationship. Given the ubiquity of intimate relationships among college students, conflict within some of these relationships is to be expected. However, a large number of college students often deal with hurt in love in an unhealthy way. Forgiveness is an important part of intimate relationships (Sheldon and Antony, 2019), and forgiveness can be an effective intervention in a relationship (Rye and Pargament, 2002). Love forgiveness has the potential to defuse relationship conflicts and improve the quality of life of college students, making it an exigent topic of investigation.

In studies by Liu (2017) and Yu (2019), Chinese male students scored higher in love forgiveness than female students. Among Chinese newlyweds, husbands show significantly higher levels of forgiveness than wives (Xie et al., 2012). This is in line with existing research on marriage in western countries: namely, that husbands were more likely to choose to forgive their wives (Fincham et al., 2002). However, Sidelinger and Booth-Butterfield (2007) claim an equal possibility for men and women to forgive each other. Nevertheless, in China, the research of Zhang and Fu (2013) showed that in romantic relationships, female students were more likely to forgive than male students. One possible reason for the inconsistencies was that in their study, male students were less satisfied with their romantic relationships than female (Zhang and Fu, 2013). The research of Liu (2017) found that love satisfaction was positively correlated with love forgiveness in China. Thus, the higher the relationship satisfaction, the higher the capacity for love forgiveness. Besides, the research of Wang and Zhao (2017) and Yu (2019) showed that Chinese male college students are more satisfied with romantic relationships than female students. From this, we speculated that male college students have more ability of love forgiveness because they have higher satisfaction with love. In addition, in the research of Zhang and Fu (2013), among the four dimensions of love forgiveness, Chinese college students score the highest in the dimension of negative thinking, while they score the lowest in the dimension of revenge, though the study of Yu (2019) showed an inconsistent result. In the love forgiveness questionnaire, Chinese college students scored the highest in the avoidance dimension, while the lowest in the forgiveness dimension (Yu, 2019).

There are also inconsistencies within prior conclusions about the demographic variables of subjective well-being and interpersonal relationships among college students. Chen (2011) found that the factors affecting the subjective well-being of college students include gender, grade, and place of origin. However, other studies showed that there is no significant gender difference in subjective well-being among college students (Cui et al., 2015). Studies have shown that there are differences in interpersonal relationships among college students in terms of grade and place of origin (Feng, 2004; Liu and Wang, 2006; Gan et al., 2007). Nevertheless, in the research of Gan and Li (2010), interpersonal relationships do not show differences in different places of origin. Interpersonal relationships had no statistically significant dependence on grade and place of origin (Gan and Li, 2010). Furthermore, Feng (2004) claims that there is no gender difference in interpersonal relationships among college students. Other studies have shown that there are gender differences in interpersonal relationships among college students, and some studies have suggested that female students have better interpersonal relationships than male students (Gan et al., 2007; Shen et al., 2007). Liu and Wang (2006) claims that male students are more agreeable than female students.

Concordant results have been obtained on the relationship among forgiveness, subjective well-being and interpersonal relationships among college students in the eastern and western cultural backgrounds. However, under the collectivist cultural background of China, there is a lack of research on forgiveness in intimate relationships. The factors affecting college students’ forgiveness in intimate relationships are still unknown. The results of studies on forgiveness in intimate relationships also need to be compared across cultures. The relationship between interpersonal relationships, subjective well-being, and college students’ love forgiveness still needs to be explored. At the same time, the existing studies on college students’ love forgiveness, subjective well-being, and interpersonal relationships have reached inconsistent conclusions on demographic variables. Hence, we will discuss whether the two factors of interpersonal relationships and subjective well-being are related to college students’ love forgiveness, as well as statistical differences among the three demographic variables of gender, grade, and place of origin.

In summary, the main goal of this study is to investigate the love forgiveness of Chinese college students and its influencing factors. Hence, we focus on the following research questions that have not been explored:

*Research Question 1*: What is the relationship between love forgiveness, interpersonal relationships, and subjective well-being of Chinese college students?
*Research Question 2*: Are interpersonal relationships an intermediary between love forgiveness and subjective well-being of Chinese college students?
*Research Question 3*: Is there any difference in love forgiveness, interpersonal relationships, and subjective well-being among Chinese college students? Are male or female college students more likely to forgive?
*Research Question 4*: Does grade level have an effect on these three variables?
*Research Question 5*: Does place of origin have an effect on these three variables?

According to the research questions, we propose the following research hypotheses:

*Hypothesis 1*: There will be correlations between love forgiveness, interpersonal relationships, and subjective well-being of Chinese college students.
*Hypothesis 2*: Interpersonal relationships will play an intermediary role between love forgiveness and subjective well-being of Chinese college students.
*Hypothesis 3*: Significant differences in love forgiveness, interpersonal relationships, and subjective well-being among Chinese college students will appear according to gender. Compared with female students, male students will score higher in love forgiveness.
*Hypothesis 4*: Among the three questionnaires and scales, significant differences will appear according to grade level.
*Hypothesis 5*: Among the three questionnaires and scales, significant differences will appear according to place of origin.

A longitudinal study of the relationship between the three variables can not only eliminate individual differences, but also study the causal relationship between the three variables. However, due to the limitation of time, energy and economy, we can only adopt cross-sectional study. The results of cross-sectional studies are only possible mechanisms, which requires readers caution and sufficient attention.

## Materials and Methods

### Participants

We took Chinese college students who are in a romantic relationship or who have ever been in a romantic relationship as the investigation objects, and selected the subjects by random sampling. Questionnaires were distributed in the libraries and classrooms of universities in China. A total of 761 paper questionnaires and 179 online questionnaires were distributed; 109 invalid questionnaires were excluded; and 831 questionnaires containing valid data were obtained, of which there were 412 male and 419 female respondents. Regarding grade level, the subjects included 286 freshmen, 159 sophomores, 192 juniors, and 194 seniors. In terms of geography, 468 subjects came from urban areas and 363 from rural areas.

### Measures

In this study, three instruments were used to explore the relationship among college students’ love forgiveness, interpersonal relationships, and subjective well-being: The College Students’ Love Forgiveness Questionnaire (CS-LFQ), College Students’ Interpersonal Relationship Comprehensive Diagnostic Scale (CS-IRCDS), and College Students’ Subjective Well-being Questionnaire (CS-SWQ). These three scales were all based on Chinese college students.

### College Students’ Love Forgiveness Questionnaire

There are 27 questions in the CS-LFQ compiled by Zhang and Fu (2014), which is divided into four dimensions: revenge, avoidance, forgiveness, and negative contemplation. Using a 6-level score (with 1 being completely consistent, 6 being completely inconsistent), forgiveness is scored opposite to the other three dimensions. The forgiveness dimension has reverse questions which are factored into the total score. The internal consistency α coefficients of the four dimensions were 0.735, 0.862, 0.877, and 0.892, respectively. The internal consistency coefficient of the total questionnaire is α = 0.897. The structural validity and external validity of the questionnaire were well. The CS-LFQ has been used by the studies of Liu (2017), Sun et al. (2018), and Yu (2019), which had good results.

### College Students’ Interpersonal Relationship Comprehensive Diagnostic Scale

Zheng et al. (2005) compiled the CS-IRCDS, which has 28 questions, including 4 dimensions: communication, conversation, dealing with people, and interacting with the opposite sex. The score in each dimension is either 0 or 1, with a 0 indicating non-conformity and a 1 indicating conformity. After the reverse questions are scored, the subject’s interpersonal relationships are measured by the total score across 4 dimensions: 15–28 points indicating minimal interpersonal troubles; 9–14 points indicating some degree of difficulty in getting along with friends; or 0–8 points indicating trouble getting along with friends. The lower the score, the more difficult it is for an individual to communicate. The reliability indexes of each subscale of the questionnaire meet the requirements of psychometric standard. The internal consistency coefficient of the total score of the scale was α = 0.82. The content validity, calibration validity and structure validity of the questionnaire were well. The CS-IRCDS has been used by the studies of Cui et al. (2015), Jiang and Tan (2016), and so on, which had good results.

### College Students’ Subjective Well-Being Questionnaire

The CS-SWQ has 61 questions and includes eight factors compiled by Jiang and Yang (2008). The questionnaire adopts a 5-level score (with 1 being completely inconsistent, 5 being completely consistent). Higher scores correspond to higher levels of subjective well-being. In the total table, the cronbach’s alpha coefficient is 0.959, and the alpha coefficient of each factor falls within the range from 0.765 to 0.916. The content validity, construct validity and calibration validity of the questionnaire were well. The CS-SWQ has been used by the studies of Jiang and Bai (2009), Jiang and Tan (2016) and so on, which had good results.

### Procedure

The CS-LFQ, CS-IRCDS, and CS-SWQ were bound into a book and responded to by college students during their self-study or rest. The subjects were first asked if they had been in a relationship and if they were willing to participate in the survey. If the students had relationship experience and were willing to participate, they were then asked to fill out the questionnaire. The questionnaire lasted about 20 min and was conducted anonymously. After each questionnaire was collected, it was checked and screened, with valid questionnaires selected for input.

This study was approved by the Ethical Committee of the School of Psychology, Nanjing Normal University. All human participants in the study were informed and consented to participate.

### Statistical Analysis of Data

The data obtained in this study were statistically analyzed by SPSS20.0 and Mplus8.3 software, and the statistical methods used are mainly descriptive statistics: *t*-test, *F*-test, and a structural variance model.

## Results

### Descriptive Statistics

#### Reliability Analysis

The internal consistency coefficients of the three questionnaires were 0.861, 0.804, and 0.961. Thus, the reliability of these questionnaires was relatively high, and the data were considered reliable.

#### Difference Analysis

We conducted multivariate analysis of variance for questionnaires and demographic variables, but the homogeneity test result of variance was significant (*F* = 4.401, *p* < 0.001), which indicated that multivariate analysis of variance was not suitable. Therefore, we performed one-way analysis of variance and *t*-test on different questionnaires.

Table 1 shows the average score and standard deviation of subjects of different gender in the CS-LFQ, CS-IRCDS, and CS-SWQ. SPSS20.0 was used to conduct independent sample *t*-tests for the average score and standard deviation of male and female samples. The results showed that in the gender difference was significant in the CS-LFQ (*t* = 6.26, *p* < 0.001, *Cohen’s d* = 0.44), in the CS-IRCDS (*t* = 3.35, *p* < 0.01, *Cohen’s d* = 0.23), and in the CS-SWQ (*t* = 5.96, *p* < 0.001, *Cohen’s d* = 0.41). The *Cohen’s d* values in here are small, which should have careful attention. Meanwhile, in the three questionnaires, the average scores of male students were higher than that of female students. Thus, the conclusion is that male students had better love forgiveness, subjective happiness, and interpersonal relationships than female students.

**TABLE 1:** Mean and standard deviation of the three questionnaires in demographic variables.

	Gender		Grade				Region	
	Male	Female	Freshman	Sophomore	Junior	Senior	City	Country
Love forgiveness	76.21±15.25	70.03±13.15	71.93±12.27	70.55±12.14	80.33±12.21	69.75±13.01	73.77±16.19	72.23±12.08
Interpersonal relationships	19.72±5.14	18.51±5.21	18.75±4.82	18.74±4.89	20.03±5.02	19.03±6.05	19.25±5.35	18.93±5.01
Subjective well-being	228.57±38.75	213.62±33.39	221.08±33.86	209.01±30.89	236.15±46.08	215.84±30.06	222.92±39.19	218.59±33.58

Table 1 shows the average score and standard deviation of subjects within different grades in the three questionnaires. SPSS20.0 was used to conduct a single factor analysis of variance to examine the average score and standard deviation of the four grades. The results showed that grade had a significant impact on results in the CS-LFQ [*F*(1,830) = 23.19, *p* < 0.001, η^2^ = 0.08], in the CS-IRCDS [*F*(1,830) = 2.76, *p* < 0.05, η^2^ = 0.01], and in the CS-SWQ [*F*(1,830) = 18.79, *p* < 0.001, η^2^ = 0.06].

In the CS-LFQ, the average score of the samples of the four grades was lowest in seniors and highest in juniors, that is, juniors had the best love forgiveness and seniors had the worst love forgiveness. In the CS-IRCDS, the sample scores of junior students were significantly higher (indicating better interpersonal relationships) than those of other grades. In the CS-SWQ, the sophomore sample scored the lowest, while the junior sample scored the highest. Thus, juniors had the highest subjective well-being and sophomores had the lowest.

Table 1 shows the average score and standard deviation of subjects at different places of origin in the three questionnaires. SPSS20.0 was used to conduct an independent sample *t*-test for the average score and standard deviation of urban and rural samples. There were no significant differences in love forgiveness, interpersonal relationships, and subjective well-being among college students from different regions. However, the scores of urban college students in the three questionnaires were slightly higher than those in rural areas, suggesting that the love forgiveness, subjective well-being, and interpersonal relationships of urban college students may be slightly better than those from rural areas.

Table 2 shows the mean and the standard deviation of different genders in the dimensions of negative contemplation, avoidance, revenge, and forgiveness in the CS-LFQ. The score of college students of different genders in different dimensions was the total score of dimensions divided by the number of items. SPSS20.0 was used to conduct independent sample *t*-test for the average score and standard deviation of male and female samples. The results revealed that there were significant differences between different genders in the four dimensions of the CS-LFQ. The *Cohen’s d* values in Table 2 are small, which should have careful attention. Male students scored higher than female students in all four dimensions. Male students scored the highest in the avoidance dimension and female students scored the highest in the negative meditation dimension.

**TABLE 2:** Gender difference test in four dimensions of love forgiveness.

	Gender	*N*	*Mean*	*SD*	*t*	*Sig.*	*Cohen’s d*
Negative meditation	Male	412	19.98	4.107	3.046**	0.002	0.212
	Female	419	19.12	4.089			
Avoidance	Male	412	20.67	6.485	5.119**	0.000	0.356
	Female	419	18.44	6.074			
Revenge	Male	412	15.33	5.235	5.417**	0.000	0.376
	Female	419	13.42	4.940			
Forgiveness	Male	412	20.23	5.938	3.001**	0.003	0.208
	Female	419	19.05	5.319			

“Annotation: **p < 0.01”.

### Relationship Research

#### Correlation Analysis

We used SPSS20.0 to convert the total scores of the three questionnaires to a standard *Z*-score, and then conducted correlation analysis. The results of this analysis showed that the three variables were correlated in pairs, as shown in Table 3.

**TABLE 3:** Correlation analysis.

	Love forgiveness	Interpersonal relationship	Subjective well-being
Love forgiveness	1		
Interpersonal relationships	0.365**	1	
Subjective well-being	0.570**	0.570**	1

Annotation: **p < 0.01.

The correlation coefficient between the CS-LFQ and CS-IRCDS was *r* = 0.37, a low correlation according to the standard. This finding indicates a weak relationship between forgiveness in a romantic relationship and the quality of interpersonal relationships. The correlation coefficient between the CS-LFQ and CS-SWQ was *r* = 0.57, a moderate correlation, indicating that the more inclined college students are to forgive in romantic relationships, the higher their subjective well-being would be. The correlation coefficient between the CS-IRCDS and CS-SWQ was *r* = 0.57, a moderate correlation, indicating that the better the interpersonal relationships of college students, the higher their subjective well-being would be. Thus, love forgiveness, interpersonal relationships, and subjective well-being of college students were all partially correlated.

#### Intermediate Inspection

Love forgiveness, subjective well-being, and interpersonal relationships were closely related among college students. According to the research hypothesis, in order to further explore the relationship among the three variables, we used Mplus8.3 to test the degree of fit of the mediating effect model of love forgiveness, subjective well-being, and interpersonal relationships among college students.

**FIGURE 1:**
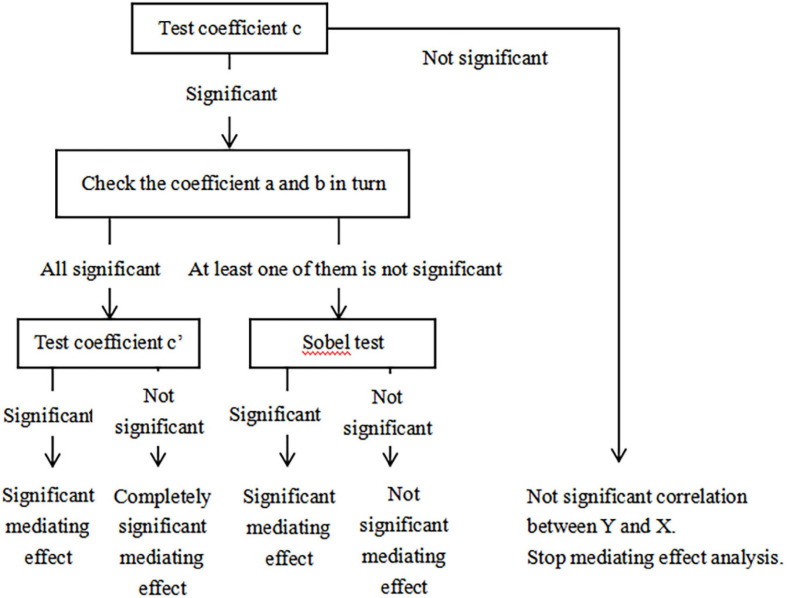
Mediating effect test method.

**FIGURE 2:**
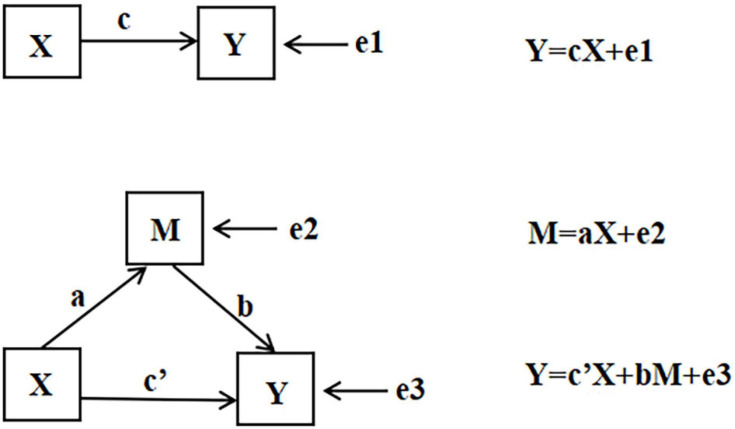
Schematic diagram of mediating variables.

On the basis of the mediation effect test method proposed by Wen et al. (2004), as shown in Figure 1, the mediation effect test in this study was carried out in steps. Figure 2 illustrates what a, b, c, and c’ represent in Figure 1.

**FIGURE 3:**
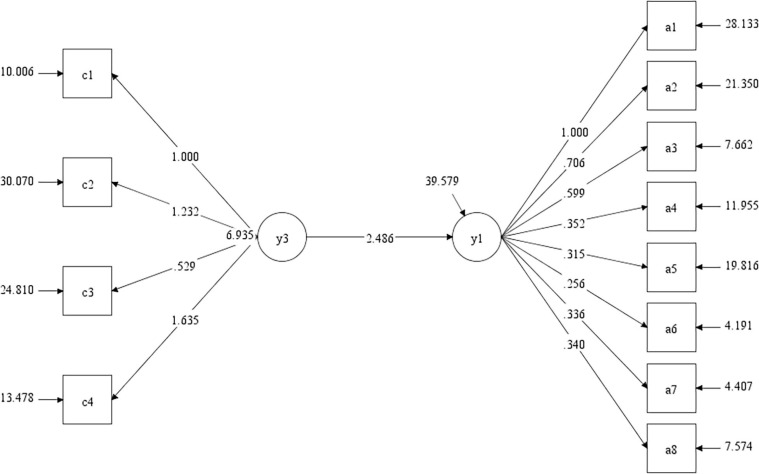
Total effect test.

Due to the good reliability of the three questionnaires, we first conducted the mediation model test by taking the total scores of the CS-LFQ and CS-SWQ as explicit variables according to the intermediary test steps, testing the direct effect of college students’ love forgiveness on subjective well-being. The test results are shown in Figure 3, with *t* = 13.94 and *p* < 0.001. The values at the upper and lower bound of each confidence interval did not contain 0, indicating that the regression coefficient C is significant.

**FIGURE 4:**
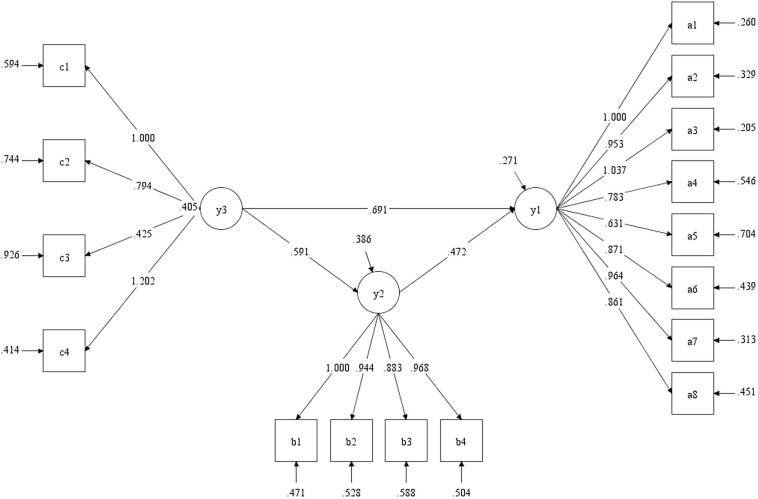
Mediating effect test.

Next, the model test of three variables was performed. The four dimensions of the CS-LFQ were taken as the latent variables of college students’ love forgiveness, the four dimensions of the CS-IRCDS as the latent variables of interpersonal relationships, and the eight dimensions of the CS-SWQ as the latent variables of subjective well-being. Taking love forgiveness of college students as the independent variable, interpersonal relationships as the intermediary variable, and subjective well-being as the dependent variable, the model was constructed. The fitting index result of the mediation model was *X*^2^ = 1170.676, *df* = 101, *p* = 0.000, RMESA = 0.113, SRMR = 0.060, CFI = 0.852, TLI = 0.824. The CFI and TLI indexes were slightly lower than 0.9, but the SRMR value is less than 0.08, indicating that there was no wrong setting in the model, but that the fitting index was not optimal. The model was not optimal and there is space for optimization, which requires caution and sufficient attention. There are two reasons for this. First, for the convenience of data processing, we treated the questionnaire scores as continuous variables, and the results obtained were only approximate values. Second, the sample size was not large enough: ideally, this fit would be performed on a sample of more than 1,000 individuals. The path diagram of mediating effect is shown in Figure 4. All the path coefficients reached the significance level, that is, the regression coefficients A, B, and C were all significant. The upper and lower limits of each confidence interval did not include 0, so the model was established and the mediating effect was significant. According to the path analysis chart, the direct effect of love forgiveness on the subjective well-being of college students is 0.69, and the mediating effect is 0.28. The overall effect of love forgiveness on subjective well-being of college students, indirect effect and direct effect reach the level of significance (*p* < 0.001). Therefore, interpersonal relationship has a significant mediating effect between these two variables. The mediating effect accounts for 40.25% of the total effect. Thus, interpersonal relationships play a mediating role between love forgiveness and subjective well-being of college students.

#### Moderator Inspection

On account of the low mediating effect of interpersonal relationship between love forgiveness and subjective well-being of college students, the model fitting index was not perfect. We used Mplus8.3 to test the moderating effect of interpersonal relationship on love forgiveness and subjective well-being of college students. The fitting index result of the mediation model was *X*^2^ = 1395.491, *df* = 146, *p* = 0.000, RMESA = 0.101, SRMR = 0.069, CFI = 0.834, TLI = 0.805. The results showed that the product item of interpersonal relationship and love forgiveness did not reach significance (*t* = 1.712, *p* = 0.087). Therefore there was no moderating effect of interpersonal relationship between love forgiveness and subjective well-being of college students. In addition, we used Mplus8.3 to the moderating effect of gender on love forgiveness and subjective well-being of college students. The fitting index result of the mediation model was *X*^2^ = 1250.280, *df* = 126, *p* = 0.000, RMESA = 0.147, SRMR = 0.094, CFI = 0.808, TLI = 0.799. The results showed that the difference between boys and girls did not reach significance (*t* = 0.953, *p* = 0.341). Therefore there was no moderating effect of gender between love forgiveness and subjective well-being of college students.

## Discussion

We investigated the demographic variables of college students’ love forgiveness, subjective well-being, and interpersonal relationships, and discussed the relationship between these three variables. We found that college students’ love forgiveness both directly and indirectly affects their subjective well-being through interpersonal relationships.

### Theoretical Implications

We found that college students’ love forgiveness, interpersonal relationships, and subjective well-being were significantly correlated; hence, the first hypothesis was valid. Love forgiveness is also a kind of forgiveness, and this result further confirms the conclusions of Jiang and Tan (2016) and Wang (2019). The results showed that the higher levels of love forgiveness corresponded to lower interpersonal distress, which is consistent with the results of Li et al. (2010). Stronger love forgiveness was linked to higher subjective well-being, which is also consistent with the results of previous studies (Thompson et al., 2005; Wang, 2014). College students with stronger interpersonal relationships also had higher subjective well-being, supporting the conclusion of He (2013).

We found that interpersonal relationships played a part in mediating the effect between love forgiveness and subjective well-being of college students, confirming hypothesis 2. Our results showed that college students’ love forgiveness had a direct impact on their subjective well-being. With the addition of interpersonal relationships, college students’ love forgiveness indirectly affected their subjective well-being through interpersonal relationships, with interpersonal relationships playing an intermediary role. For example, when college students show the tendency or behavior of forgiveness in an intimate relationship, their positive emotions increase and negative emotions decrease, thus improving their subjective happiness. As another example, college students who show the tendency or behavior of forgiveness in love can better establish and maintain their interpersonal relationships in daily life, have less interpersonal troubles, and thus improve their subjective well-being.

Jiang and Tan (2016) found that interpersonal relationships mediate the forgiveness and subjective well-being of college students. Bono et al. (2008) and Tse and Yip (2009) found that the intimate relationship between the offender and the offended is the main factor affecting the subjective well-being of forgiveness. On the basis of previous studies, this study deeply explored the relationship between college students’ forgiveness in an intimate relationship—systematized as love forgiveness—and other variables. We also found that interpersonal relationships play a partial mediating role between love forgiveness and subjective well-being of college students. This not only expands the research in the field of forgiveness, but also enriches our understanding of forgiveness in intimate relationships, filling the research gap of love forgiveness in the context of Chinese collectivist culture.

We found significant differences in the CS-LFQ, CS-SWQ, and CS-IRCDS according to gender. Male students scored higher in the CS-LFQ than female students. Therefore, hypothesis 3 was valid. Studies showed that male college students demonstrated more forgiving tendencies or behaviors than female college students when it comes to hurt in romantic relationships. This is consistent with the Chinese research results of Liu (2017) and Yu (2019), as well as the Chinese research conclusions of Xie et al. (2012) on marital relations, namely, that husbands are more likely to choose to forgive their wives. This result that may be reflective of cultural norms in China, where male are dominant and female are dependent in the process of love (He et al., 2008). He et al. (2008) believed that under this mode, Chinese male have a higher sense of relationship satisfaction, which is different from the relevant research result in the Western countries (Kurdek and Schmitt, 1986). Furthermore, the Chinese research of Liu (2017) found love satisfaction was positively correlated with love forgiveness, which was consistent with the results of Western studies on marriage. The Western studies showed that marital satisfaction is a significant predictor of marital forgiveness (Fincham and Beach, 2002; Fincham et al., 2004, 2007). Meanwhile, the research of Wang and Zhao (2017) and Yu (2019) confirmed that male college students are more satisfied with love than female college students. Therefore, Chinese male college students have better love forgiveness ability than female college students, because they have higher love satisfaction in the Chinese cultural context. Then, both male and female college students scored lowest in the retaliation dimension, which was consistent with Zhang and Fu (2014). College students are less likely to choose revenge in romantic relationship, this may be reflective of Chinese cultural norms, which derive from a tradition of Eastern collectivism again. Ye (2004) pointed out that collectivist groups are not good at anger and tend to suppress anger in order to maintain a good interpersonal relationship, that is, to form a negative internal meditation. Some Chinese studies had also pointed out that collectivist individuals have their own unique forgiveness mechanism. After being hurt, they are unwilling to choose aggressive coping methods out of maintaining harmonious relationship and caring about face (Tao and Fu, 2010; Zhang et al., 2012). In addition, male students scored highest in the avoidance dimension, meanwhile female students scored highest in the negative meditation dimension. However, in the study of Zhang and Fu (2014), both male and female students scored highest on negative meditation. Negative contemplation is one of the coping methods of Chinese college students when they face the harm of love (Zhang and Fu, 2014). The study had pointed that male college students in contemporary China lack the sense of distress and responsibility, but they hope to maintain the traditional male dominant position in emotional communication and marriage life, which is directly related to the unique Chinese “son over daughter” old thought of family concept (Chen, 2013). This may be the cause of male college students’ ambivalence and avoidance in the face of love problems or injuries.

College students’ subjective well-being, dependent on their scores in the CS-SWQ, was generally at a high level, with a significant difference between male and female. Chen (2011) and Liu and Liu (2014) pointed out that gender differences in subjective well-being are related to gender role expectations. However, the subjective happiness of female college students was lower than that of male college students, a finding that is inconsistent with previous results (Cui et al., 2015; Wang, 2019). The possible reason is that with the progress of Chinese society, women have been given more responsibilities than before, and women have to take care of their careers while taking care of their families. Whether in school or in the workplace, women are often treated less equally than men. As a result, women face more stress, resulting in lower subjective well-being than men. There was a significant gender difference in the interpersonal relationships of college students, and male students were indicated as easier to get along with than female students, consistent with the conclusion of Liu and Wang (2006). Liu and Wang (2006) found that the reason why males have better interpersonal relationships than females is that females have more interpersonal problems than males.

We found significant differences between grade levels in the CS-LFQ, CS-SWQ, and CS-IRCDS, confirming the validity of hypothesis 4. College students’ love forgiveness showed significant differences in grades, which is consistent with the research results of Li et al. (2010) on college students’ forgiveness. Junior year is the year with the highest level of romantic forgiveness and senior year is the year with the lowest romantic forgiveness, according to our results. This is consistent with the research conclusion of Xu and Liang (2013), the forgiveness level of contemporary college students shows a fluctuating trend. However, in the research of Li et al. (2010), the forgiveness level was highest in the first year and lowest in the third year, which was inconsistent with our results. This difference may be due to the object of forgiveness. The research has shown that the object of forgiveness is one of the factors that influence forgiveness Xiong (2014) and Yun and Guo (2018) indicated that forgiveness is more likely to occur in closer and more committed relationships in the meantime. Specifically, love forgiveness specifically refers to the forgiveness of the other one in the intimate relationship, while forgiveness involves more objects and events of forgiveness. Therefore, the level of love forgiveness of college students may not be consistent with the level of forgiveness. With the growth of age and the increase of love experience, college students gradually become mature, better at dealing with love problems and the level of love forgiveness increases. However, the future of college students has been decided in the senior year, the couples is faced with the test, whether to continue to be together or face reality to break up. In order to provide a reason for breaking up, it may be that some previously forgivable problems become unforgivable. As a result, the level of love forgiveness was lowest among college students in senior year and was highest in the junior year. More researches are needed to explore the mechanisms of love forgiveness in college students.

The research showed that there are significant differences in the subjective well-being of college students, and the subjective well-being index of sophomore year was the lowest, junior year was the highest, which is consistent with the research results of Liu and Liu (2014). The research showed that there are significant differences in the interpersonal relationships of college students, and the interpersonal relationship of higher grade is better than that of lower grade, which is also consistent with the results of previous studies (Li et al., 2010; Wang, 2015).

We found that the place of origin of students didn’t show significant differences in the three questionnaires and scales; hence, hypothesis 5 was not valid. Although there was no significant difference among the three questionnaires according to place of origin, the love forgiveness, subjective well-being, and interpersonal relationships of college students from urban areas were slightly better than those from rural areas. The reason for the insignificant difference may be that the sample is not well represented. The sampling area is Jiangsu Province, China, which is a relatively economically prosperous region in China. The gap between urban and rural areas is relatively small. At the same time, China’s urbanization process is accelerating and the gap between urban and rural areas is narrowing. Therefore, the difference between college students from different places of origin is not obvious enough. Thus, we anticipate the need to increase the sample size or collect data from multiple regions for more detailed studies.

### Practical Implications

We found that interpersonal relationships and subjective well-being are correlated with college students’ love forgiveness. Hence, in the psychological counseling of college students about interpersonal relationships or subjective well-being, the psychological treatment of forgiveness intervention can be considered to improve their interpersonal relationships or subjective well-being through guiding the forgiveness behavior of college students. The object of forgiveness should also include the object of love. In order to avoid the increase of interpersonal troubles and the decrease of subjective happiness, counselors can guide students in facing potential harms caused by romantic relationships, with a focus on helping them learn forgiveness. When students are hurt in a romantic relationship, the love forgiveness framework presents four reactions: negative meditation, avoidance, revenge, and forgiveness. We found that the majority of college students chose forgiveness and negative meditation. This provides a new way of thinking for psychological counselors to approach the intimate relationship problems of college students. Given college students who adopt different coping styles in romantic relationships, psychological counselors can use the love forgiveness framework to develop counseling programs to provide the most effective and targeted help to college students.

### Limitations

The strengths of this study is that it reveals the relationship between college students’ interpersonal relationship, subjective well-being and love forgiveness, and provides a new idea for counselors to deal with college students’ intimate relationship problems. However, this study has the following limitations:

(1) As the SEM was applied, the error generated by the measurement process was included in the analysis process while exploring the relationship between the variables.

(2) The subject population may not be representative. There are two reasons. One is that the subjects selected for this study came from Jiangsu Province, which is an economically prosperous region in China. One is that the sample size is not large enough. If the sample size is increased to more than 1,000, the obtained model data may be better.

(3) This study was a cross-sectional study. The results indicated a possible mechanism and did not prove a causal relationship, which should need readers caution attention.

(4) Participants responded to questionnaires based on the most damaging event their partner had done to them in romantic relationship. Love forgiveness is forgiveness within a specific relationship (Zhang and Fu, 2014). Therefore, the harm suffered in the romantic relationship is also the harm within a specific relationship, which is distinct from general harm. Even in a romantic relationship, people experience different kinds of damage. However, this study did not categorize the type and nature of the injuries.

### Directions for Future Research

Above all, the factors influencing college students’ love forgiveness are complex and diverse. Social cognition, personality traits, mental health, culture, and other factors all affect forgiveness. The question of whether these factors affect love forgiveness in the same way provides an avenue for follow-up research. Whether the difference between college students from urban and rural areas has an impact on their love forgiveness, subjective well-being, and interpersonal relationships has not been established yet. Lastly, there is a lack of research on forgiveness in marital relationships in China. Further studies could establish whether there is a difference between love forgiveness in marital relationships and in non-marital romantic relationships, which can also be compared across cultures.

## Data Availability Statement

The original contributions presented in the study are included in the article/Supplementary Material, further inquiries can be directed to the corresponding author/s.

## Ethics Statement

This study was approved by the Ethical Committee of the School of Psychology, Nanjing Normal University. All human participants in the study were informed and consented to participate.

## Author Contributions

TC completed the data collection, article writing, and modification and translation work. QL gave important guidance to the manuscript. HF provided guidance on the revision of the manuscript. All authors contributed to the article and approved the submitted version.

## Conflict of Interest

The authors declare that the research was conducted in the absence of any commercial or financial relationships that could be construed as a potential conflict of interest.
