# Supplementary material for: Love Forgiveness and Subjective Well-Being in Chinese College Students: The Mediating Role of Interpersonal Relationships
Source: Front Psychol. 2021 Jun 4;12:634910. doi: 10.3389/fpsyg.2021.634910 (PMC8211753; doi:10.3389/fpsyg.2021.634910)
Supplement: Supplementary file 1 [file Data_Sheet_1.PDF]

Dear students：

Hello!

Thank you very much for taking part in this survey, which is divided into three parts. This is a questionnaire about college students' love, interpersonal, life and so on. This survey is anonymous. Your answers will only be used for this research and will be kept confidential to others. Please feel free to fill in. Please be careful not to omit or not answer or choose more than one question, and do not misplace the question. There are no right or wrong, good or bad answers to these questions. Please read each one carefully and answer independently according to your actual situation.

(Note: If any of the questions in the list has not happened before, please complete it according to your hypothesis or deduction.) Although there is no time limit, it should be done as quickly as possible. Thank you for your cooperation, wish you a happy life!

Gender: _____________ professional:_____________

Age: _____________ grade:_____________

Have you ever been in a relationship:_____________

Registered Place (urban/rural) :_____________

Part One

Please judge the degree of each of the following questions according to your actual situation, and tick √ in the corresponding ( ).

		Totally not conform	Basically not conform	Not sure	Mostly conform	Fully conform
1	I always find the class does not understand, there is no spirit.					
2	I have a lot of employment-related issues that get on my nerves.					
3	On the whole, there are more happy things in my life than unhappy things.					
4	I often feel depressed.					
5	I am interested in my major.					
6	I'm always optimistic about the future.					
7	I feel my life is full.					
8	I always have confidence in myself.					
9	I often feel empty and lonely.					
10	I always feel the study task is heavy, the study pressure is very big.					
11	I think people are always mercenary to each other.					
12	I often feel the dark side of life.					
13	I'm not sure of the path I want to take in the future.					
14	In general, I can get the care of my lover.					

15	My life is generally satisfactory.					
16	I am interested in many things in life.					
17	I often feel depressed.					
18	I have insurmountable obstacles in my studies.					
19	In general, I have the support of my lovers.					
20	I'm generally happy with myself.					
21	I have my own goals and work hard for them.					
22	I'm always optimistic.					
23	Things in life always make me tired.					
24	My academic performance can be recognized.					
25	When I think about employment, I often feel apprehensive.					
26	In general, I can get the understanding of the lover.					
27	I’m not satisfied with the way I look.					
28	College life has its ups and downs, but on the whole it is satisfactory.					
29	I feel my life is very meaningful.					
30	I always feel lonely.					
31	On the whole, I am satisfied with my studies.					
32	I always get care from my friends.					
33	My relationship with my lover is good and stable.					
34	I think there are always a lot of things in life to make people depressed.					
35	I always have low spirits.					
36	I am quite satisfied with the statement of my study.					
37	On the whole, I have a positive attitude towards myself.					
38	I can always get my friends to understand me.					
39	I think society is very unfair.					
40	I think the employment problem in the future is very stressful to myself.					
41	I can get approval from others.					
42	I have clear learning goals.					
43	I always think other people are better than me.					

44	I can always get support from my friends.					
45	I'm worried about my life in the future.					
46	I always feel less capable than others					
47	I don't have too many complaints about life.					
48	I'm always at a disadvantage in the competition.					
49	The courses I am studying are very taxing.					
50	I always feel idle.					
51	I am optimistic about my employment prospects.					
52	I always feel like I'm out of my depth.					
53	I always have too many complaints about life.					
54	I often feel that my efforts are rewarded.					
55	I always feel like I'm wasting my time.					
56	My major is boring					
57	I was annoyed and bored by many of the phenomena around me.					
58	I'm always doubting whether I'll ever find a proper job.					
59	In general, I am happy emotionally.					
60	I often feel lucky.					
61	I often feel helpless.					

Part Two

Please recall the most hurtful thing your current or former partner did to you in romantic relationship. How would you react to a similar injury in a relationship? The following are some of the reactions that people often have when facing injury. Please choose the degree to which the following reactions correspond to you according to your actual situation and tick √ in the corresponding ( ).

		Fully conform	Basically conform	Kind of conforming	Kind of not conforming	Basically not conform	Totally not conform
1	Find ways to make him or her feel guilty.						
2	Find a way to get back what I lost.						
3	Do something that hurts him or her more.						
4	Take revenge on him or her.						
5	Keep your distance from him or her.						
6	Don't want to have anything to do with him or her.						
7	Stop trusting him or her.						
8	Don't feel comfortable around him or her.						
9	End the relationship.						
10	Be willing to forgive him or her.						
11	Treat him or her as before.						
12	Quickly forget the harm he or she to myself.						
13	Make up again quickly.						
14	Sulk by myself.						
15	Experiencing negative emotions such as anger.						
16	Feel in bad mood.						
17	Feel depressed.						
18	Always think about the hurt he or her did to me.						

Part Three

Please answer truthfully according to your own actual situation, make a judgment of "Yes" or "No" for each question, and tick √ in the corresponding ( ).

		YES	NO
1	I Has no words for the troubles.		
2	Meeting new people doesn't make me feel natural.		
3	I am envy and envy others excessively.		
4	I am too little interaction with the opposite sex.		
5	It is difficult to for me talk continuously.		
6	I feel nervous in social situations.		
7	I always hurt people.		
8	I don’t feel natural to interact with the opposite sex		
9	I always feel lonely or lost with a large group of friends.		
10	I easily feel embarrassed.		
11	I can't get along well with others.		
12	I don't know where it should stop,when getting along with the opposite sex.		
13	I feel usually uncomfortable when a stranger confides his life story for sympathy.		
14	I worry about what bad impression people will have of me.		
15	I always try to make others appreciate me.		
16	I secretly yearn for the opposite sex.		
17	I often avoid expressing my feelings.		
18	I have no confidence in my appearance.		
19	I dislike someone or be disliked by someone.		
20	I despise the opposite sex.		
21	I can't listen intently.		
22	My troubles have no one to talk to.		
23	I am ostracized and indifferent by others.		
24	I am looked down upon by the opposite sex.		
25	I can't listen to a wide range of opinions.		
26	I often feel sad in secret because I am hurt.		
27	I am often talked about and made a fool of.		
28	I don't know how to get along better with the opposite sex.		

Your questionnaire survey is finished. Thank you for taking time out of your busy schedule to help us carry out the survey. Thank you for your active participation! Wish you a happy study and life!
